# Unveiling the Antimicrobial Potential of Ricinus communis: A Comprehensive Review of Its Relevance to Surgical Site Infection (SSI) Pathogens

**DOI:** 10.7759/cureus.99803

**Published:** 2025-12-21

**Authors:** Alok Kumar Arya, Gaurav Kumar, Vineet SIngh, Nashra Afaq, Snehanshu Shukla, Stuti Singh, Palash Ratna, Madhu Yadav, Shikha Pandey, Atma Nand Yadav

**Affiliations:** 1 Department of Forensic Medicine and Toxicology, Rajarshi Dashrath Autonomous State Medical College, Ayodhya, IND; 2 Department of Microbiology, Rajarshi Dashrath Autonomous State Medical College, Ayodhya, IND; 3 Department of Dermatology, Venereology, and Leprosy, Maharshi Vashishtha Autonomous State Medical College, Rampur, IND; 4 Department of Microbiology, Rama Medical College, Kanpur, IND; 5 Department of Pharmacology, Umanath Singh Autonomous State Medical College, Jaunpur, IND; 6 Department of Pharmacology and Therapeutics, King George's Medical University, Lucknow, IND; 7 Department of Microbiology, Ganesh Shankar Vidyarthi Memorial Medical College, Kanpur, IND; 8 Department of Microbiology, Madhav Prasad Tripathi Medical College, Basadiliya, IND; 9 Department of Forensic Medicine and Toxicology, Era Lucknow Medical College and Hospital, Lucknow, IND

**Keywords:** antimicrobial resistance, medicinal plants, methanolic extract, phytotherapy, ricinus communis, surgical site infection

## Abstract

Surgical site infections (SSIs) remain a major global healthcare challenge, significantly affecting postoperative recovery, prolonging hospital stays, and increasing healthcare costs. The growing prevalence of antimicrobial resistance further complicates their management by limiting the effectiveness of existing antibiotics. The reduced efficacy of conventional antimicrobial agents due to resistance development, poor bioavailability, and toxic side effects has intensified scientific interest in safe, plant-based therapeutic alternatives. *Ricinus communis* (castor oil plant), a widely used medicinal species, exhibits a diverse pharmacological profile, including antibacterial, anti-inflammatory, antioxidant, and wound-healing properties. This review comprehensively synthesizes ethnobotanical, phytochemical, and microbiological evidence regarding the antimicrobial potential of *R. communis*, emphasizing its activity against bacterial pathogens frequently associated with SSIs. Evidence from global literature demonstrates that methanolic and ethanolic extracts of *R. communis* leaves exhibit potent inhibitory activity against multidrug-resistant microorganisms, including *Staphylococcus aureus*, *Escherichia coli*, *Klebsiella pneumoniae*, and *Pseudomonas aeruginosa*. These activities are primarily attributed to bioactive phytoconstituents such as ricinine, gallic acid, kaempferol, quercetin, and flavonoid derivatives. Collectively, findings from around the world identify *R. communis* as a promising phytotherapeutic candidate for infection management. However, further studies focusing on safety, pharmacokinetics, extraction standardization, and clinical validation remain essential for its integration into modern evidence-based medicine.

## Introduction and background

Epidemiology of surgical site infections

Antimicrobial resistance (AMR) is among the most significant health issues of the 21st century. It occurs when microorganisms such as bacteria, viruses, fungi, and parasites evolve mechanisms to resist the effects of antimicrobial drugs, much like weeds becoming resistant to herbicides over time. Previously curable diseases now persist longer, increasing healthcare costs and mortality [1]. The World Health Organization (WHO) has reiterated the importance of new therapeutic approaches to tackle the challenges posed by resistant pathogens, especially in healthcare-associated infections, including surgical site infections (SSIs). One of the most common nosocomial (hospital-acquired) infections, SSI, continues to be a significant cause of postoperative morbidity and mortality, extending hospital stays and increasing mortality on a global scale [2,3].

Although antibiotics continue to advance, traditional antimicrobial agents remain subject to various limitations, including a narrow therapeutic index, limited bioavailability, unfavorable pharmacokinetics, toxicity, and allergic reactions [4]. Moreover, the emergence of multidrug-resistant (MDR) bacterial strains, including *Klebsiella pneumoniae*, *Escherichia coli*, and methicillin-resistant *Staphylococcus aureus* (MRSA), many of which produce extended-spectrum β-lactamases (ESBLs), has dramatically diminished the effectiveness of available treatment options [5,6]. Consequently, traditional antimicrobial regimens are no longer sufficient to manage infections caused by resistant organisms.

Need for plant-based antimicrobials

To address these issues, the global scientific community has increasingly turned toward plant-based medicine, recognizing its potential to provide safer and more sustainable therapeutic alternatives [7]. Ethnopharmacology, a branch of pharmacology focused on traditional medicinal practices, has regained popularity as scientists explore the vast therapeutic potential of botanical species [7]. Medicinal plants are rich in bioactive compounds, known as phytochemicals (naturally occurring plant chemicals with biological activity), including alkaloids, phenolics, terpenoids, flavonoids, and glycosides, many of which possess strong antimicrobial properties [8].

*Ricinus communis* (castor oil plant) is among the most extensively studied medicinal species and has been traditionally used to treat inflammation, microbial infections, gastrointestinal disorders, and dermatological conditions [9]. Its extracts demonstrate broad-spectrum antimicrobial, anti-inflammatory, and wound-healing properties [10,11]. Research indicates that methanolic, ethanolic, and aqueous extracts of *R. communis* leaves exhibit potent inhibitory effects against pathogenic bacteria and fungi, including resistant clinical isolates [3,12]. These bioactivities are primarily attributed to phytochemicals such as ricinine, kaempferol, gallic acid, and flavonoid glycosides, which display significant activity against both Gram-positive and Gram-negative organisms [13-15]. While plant-based antimicrobials offer promising alternatives, their therapeutic use is limited by factors such as variability in phytochemical composition, lack of standardized extraction methods, and insufficient clinical validation. This highlights the need for further research to establish consistent efficacy and safety profiles.

Objective of the review

This review synthesizes existing ethnobotanical, phytochemical, and microbiological evidence on the antimicrobial potential of *R. communis*, with a specific focus on its activity against pathogens associated with SSIs. It also highlights key bioactive constituents, mechanisms of action, and existing research gaps to support the development of standardized, safe, and clinically validated phytotherapeutic alternatives.

Methodology

This narrative review followed PRISMA-adjacent principles to ensure transparency and reproducibility. A comprehensive literature search was conducted in PubMed, Scopus, ScienceDirect, and Google Scholar from January 2010 to June 2024. The search strategy used Boolean operators and controlled vocabulary terms, combining “*Ricinus communis*”, “castor oil plant”, “antimicrobial activity”, “surgical site infections”, “phytochemicals”, “pharmacological properties”, “nanoparticles”, and “multidrug-resistant bacteria”.

Inclusion criteria encompassed peer-reviewed research and review articles reporting findings on the phytochemical composition, pharmacological activities, or antimicrobial properties of *R. communis*. Studies investigating biological, toxicological, or therapeutic effects, including anti-inflammatory, antiviral, antifungal, and wound-healing activities, were also included to provide a comprehensive understanding of its biomedical potential. Both in vitro and in vivo studies were considered.

Exclusion criteria were limited to non-English publications, non-peer-reviewed sources, and studies without accessible full text or relevant biological data. Literature focusing exclusively on industrial or agricultural applications without biomedical implications was included only when it provided insight into biochemical pathways or phytochemical constituents relevant to pharmacological activity. The selected studies were qualitatively synthesized to highlight the antimicrobial efficacy of *R. communis*, its phytochemical diversity, mechanisms of action, and associated knowledge gaps.

Study selection and data synthesis

Titles and abstracts retrieved from the database search were screened manually for relevance. Duplicates were removed, and the remaining studies were reviewed in full text. The selected papers were qualitatively synthesized through narrative thematic analysis, grouping evidence into major domains, including ethnobotanical background, phytochemical composition, antimicrobial efficacy, and therapeutic implications. Emphasis was placed on reproducible findings, methodological soundness, and emerging research trends.

Risk of bias and quality considerations

As this work is a narrative review, formal quantitative risk-of-bias tools (e.g., Cochrane or ROBINS-I) were not applied. However, study credibility was ensured by including only peer-reviewed and data-driven research with clear experimental designs and verifiable methodologies.

## Review

Ethnobotanical and traditional uses of *Ricinus communis*

*R. communis* L. is a hardy perennial shrub belonging to the family Euphorbiaceae. It is widely cultivated across tropical and subtropical regions, although it originated in India, Eastern Africa, and the southeastern Mediterranean Basin [16]. The plant can grow up to six meters in height and is characterized by large, palmately lobed leaves with toothed margins and prominent venation. Its fruit is a spiny capsule enclosing oil-rich seeds that contain ricin, a toxic protein responsible for the plant’s poisonous reputation. The monoecious flowers are typically greenish-yellow with crimson stigmas [17]. Taxonomically, *R. communis* falls under the kingdom Plantae, order Malpighiales, and family Euphorbiaceae, within the genus *Ricinus* and species *R. communis*.

Despite the toxicity of its seeds, other plant parts, especially the leaves and stems, have long been recognized for their medicinal value across various traditional systems. These parts are rich in bioactive compounds, including alkaloids, flavonoids, terpenoids, phenolics, saponins, glycosides, and tannins, which contribute to their antimicrobial, anti-inflammatory, and antioxidant activities [18]. The phytochemical diversity of *R. communis* underpins its pharmacological potential, though variability in compound concentration due to geographic, environmental, and extraction factors may influence its therapeutic consistency.

Historical and traditional uses

*R. communis* has been an integral part of ethnomedicine for millennia. In Ayurveda and Unani traditions, it has been used to treat arthritis, dysentery, skin disorders, gastrointestinal ailments, and reproductive health issues [19]. In India, castor oil remains a household remedy for constipation, rheumatism, and inflammatory skin conditions. Ancient Egyptians employed it as both a cosmetic and a laxative, while African and Middle Eastern societies have used the leaves as poultices for wounds and swellings [20]. These historical applications form the foundation for its renewed scientific exploration in modern phytopharmacology.

Key phytochemicals

The main factor driving this plant's pharmacological action is its rich content of bioactive secondary metabolites. These predominant phytochemical classes are flavonoids, alkaloids, phenolics, and terpenoids [21]. The leaves and stems also contain flavonoids, including quercetin and kaempferol glycosides. Ricinine is among the largest alkaloids of the leaves and seeds, which is antimicrobial and insecticidal [22]. The phenolic compound gallic acid has been reported to possess antimicrobial, antioxidant, and hepatoprotective potential [23]. Other terpenoids found in the plant are widely used as therapeutics, including anti-inflammatory and antifertility agents [24]. More recently, a second new alkaloid, ricicomin A, has been isolated from the leaves, further expanding the plant's pharmacological toolbox and suggesting the drug will be developed [25].

Bioactive properties

This narrative review examines the antimicrobial and therapeutic effects of *R. communis*, in particular its action against bacterial species most likely to cause SSIs. These pathogens, such as *Klebsiella pneumoniae*, *Pseudomonas aeruginosa*, *Escherichia coli*, and *Staphylococcus aureus*, are significant to treat because of the multidrug resistance [17,18].

Scope of the review

The purpose of the narrative review is to present an argument on the antimicrobial and therapeutic effects of *R. communis* with respect to its ability to combat bacteria known to be the causes of SSIs. SSIs have gained notoriety due to the presence of organisms resistant to most antibiotics, including *Klebsiella pneumoniae*, *Pseudomonas aeruginosa*, *Escherichia coli*, and *Staphylococcus aureus* [17,18].

Recent studies using conventional microbiological methods, broth dilution, and disc diffusion with *R. communis* leaf extracts have shown high antibacterial activity against these pathogens [3,19]. As an example, the inhibitory activity of the experimental past was documented at 23 mm against *Staphylococcus aureus* and 19 mm against *Escherichia coli* and *Klebsiella pneumoniae*, indicating that these extracts may be used as prophylaxis or treatment for SSIs [19].

Moreover, the antimicrobial effect of *R. communis* is not the only pharmacological effect of this plant. The fact that it has anti-inflammatory [2], anti-arthritic [5], antipyretic [11], antiviral [1], antioxidant [10], and antiproliferative [7] properties makes its use in integrative medicine increase. These multitasking activities make *R. communis* a potential new development in new combinations against polymicrobial and inflammatory diseases, including SSIs.

**Figure 1:**
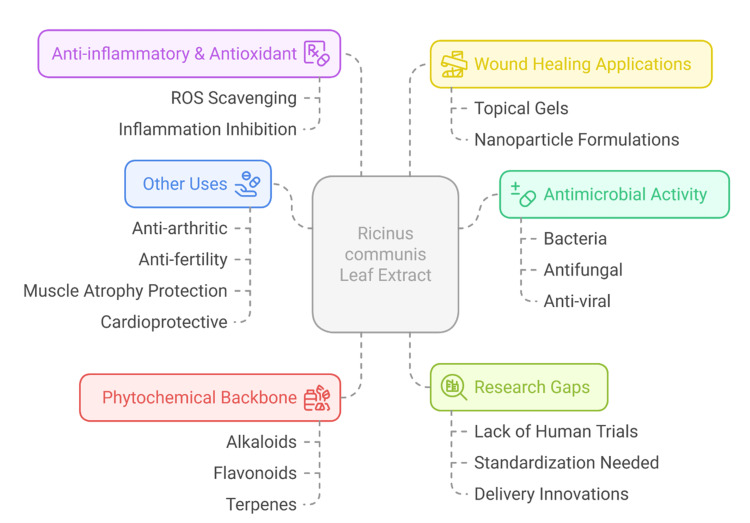
Pharmacological profile of Ricinus communis leaf extract Image Credit: Atma Nand

Considering these findings, this review will critically examine the phytochemical composition of *R. communis*, its in vitro antimicrobial effects, particularly against SSI-related pathogens, the modes of action as postulated by earlier research, and the pharmacological implications and translational applications. The review is founded on the realization that AMR requires viable bio-based solutions. The time-tested herbal medicine *R. communis* can help modern medicine return to the integrative path to combat infectious disease [26,27]. Figure 1 illustrates the various pharmacological activities, phytochemical components, and research gaps associated with *R. communis* leaf extract.

*R. communis* has the most extensive antibacterial activity and applies to both Gram-positive and Gram-negative bacteria. It has been demonstrated that its extracts can inhibit the growth of pathogens such as *Staphylococcus aureus*, *Escherichia coli*, and *Klebsiella pneumoniae*, and this has led many scientific studies to confirm its long history of clinical use [3,12]. Moreover, the plant has been shown to possess significant antidiabetic properties, increasing insulin sensitivity and glucose metabolism. Its extracts have also been shown to show anticancer properties, particularly against breast and colon cancer cell lines, where it inhibits cell growth and, in addition, triggers apoptosis [6,13].

Another field where *R. communis* has traditionally served as a natural birth control method in the reproductive sphere. Modern studies also confirm its antifertility and spermicidal effects, which can be attributed to its specific phytochemical composition [10]. The plant is endowed with a wide array of phytochemicals by way of flavonoids, alkaloids, phenolics, and terpenoids that are all related to the pharmacological action of the plant. These compounds and their bioactivities are summarized in Table 1.

**Table 1:** Key phytochemicals and bioactive properties of Ricinus communis

Phytochemical	Chemical class	Reported bioactivity	Reference
Quercetin	Flavonoid	Antioxidant, anti-inflammatory	[8]
Kaempferol	Flavonoid	Antioxidant, anticancer	[8]
Ricinine	Alkaloid	Antimicrobial, insecticidal	[9]
Gallic acid	Phenolic acid	Antioxidant, hepatoprotective	[14]
Ricicomin A	Alkaloid	Antibacterial (novel compound)	[15]
Terpenoids	Terpenoid	Antifertility, anti-inflammatory	[10]
Mixed flavonoids	Flavonoid	Antidiabetic, anticancer	[13]

Surgical site infections: epidemiology and microbial landscape

SSIs are a significant problem in contemporary healthcare, constituting about 20% of healthcare-related infections and affecting up to 5% of surgical patients worldwide [28,29]. The incidence rates are significantly different across geographical regions, health infrastructure, the nature of the surgical procedure, and the effectiveness of infection control measures [30]. In low- and middle-income nations, SSI prevalence exceeding 10% has been reported, presenting a serious risk to patient survival and postoperative outcomes [31].

Significant clinical repercussions result from SSIs. They can interfere with or postpone crucial treatments such as chemotherapy or radiation therapy, slow down wound healing, and increase hospital stays, intensive care unit admissions, and readmissions [32]. The economic burden is also substantial due to prolonged hospitalization, repeated diagnostic testing, and increased antimicrobial consumption [33].

Recent global surveillance reports indicate a rising prevalence of MDR SSI pathogens, particularly MRSA, ESBL-producing *Escherichia coli* and *Klebsiella pneumoniae*, and carbapenem-resistant Enterobacteriaceae (CRE). According to these updated findings, resistance rates in *Staphylococcus aureus* exceed 45% in several Asian and African regions. In comparison, ESBL-producing *Escherichia coli* and *Klebsiella pneumoniae* strains account for up to 60% of SSI isolates in low-resource hospitals [34]. These trends highlight the critical need for integrated antimicrobial stewardship, continuous surveillance, and the exploration of novel plant-based therapeutics for infection management.

Common pathogens in SSI cases

SSI possesses the microbiological spectrum of Gram-positive and Gram-negative bacteria. The common Gram-positive coccus responsible for staphylococcal infections is *Staphylococcus aureus*, and, more so, MRSA due to its high virulence and resistance to multiple drugs [5,30]. Other significant causes include *Escherichia coli*, *Klebsiella pneumoniae*, and Gram-negative *Pseudomonas aeruginosa*. Such pathogens can also develop biofilms, enhancing their resistance and persistence to antimicrobial management [31].

Pathogen resistance patterns

Resistance patterns of SSI pathogens are increasingly concerning. MRSA is resistant to β-lactams, aminoglycosides, and fluoroquinolones, which have few treatment options, including vancomycin and linezolid [32]. Enterobacteriaceae-producing ESBL include *Klebsiella pneumoniae* and *Escherichia coli*, which are resistant to aztreonam, cephalosporins, and penicillins. Moreover, the development of CRE also restricts the number of therapeutic options [33]. *Pseudomonas aeruginosa* has intrinsic and acquired resistance mechanisms that confer high-level resistance to multiple antibiotic classes, including aminoglycosides and fluoroquinolones, and these mechanisms include efflux pumps and enzyme production [31]. The growing prevalence of these resistant pathogens, as reported in global surveillance studies, underscores the urgency of developing novel methods to address antibacterial resistance. Past studies have shown that bioactive compounds from therapeutic plants, such as *R. communis* can serve as an alternative source of safe, effective supplements or as an alternative to conventional antibiotics.

Antimicrobial efficacy against SSI pathogens

The antimicrobial efficacy of *R. communis* has been validated across multiple regions, including Asia, Africa, and South America. Several in vitro and in vivo studies have demonstrated its ability to inhibit clinically relevant pathogens. According to published reports, extracts of *R. communis* have shown strong antibacterial and antifungal activity in Ethiopia, particularly against *Candida albicans* and *Escherichia coli* [2]. The potential of ethanolic extracts against drug-resistant microorganisms was further supported by their effectiveness against *Helicobacter pylori*, which is resistant to antibiotics [21]. In Nigeria, methanolic extracts of *R. communis* showed both cardioprotective and antimicrobial properties, suggesting that the same phytochemicals may have dual therapeutic benefits [8]. Minimum inhibitory concentration (MIC) and minimum bactericidal concentration (MBC) studies from India and Brazil reported inhibitory values as low as 1.5625 mg/mL against *Staphylococcus aureus* and *Escherichia coli*, confirming earlier disc diffusion findings reported in previous studies [30].

Several studies have specifically evaluated the antimicrobial potential of *R. communis* against pathogens commonly implicated in SSIs. Abew et al. [3] demonstrated significant inhibitory activity of leaf extracts against *Escherichia coli* and MRSA, two of the most frequent SSI pathogens. Voleti et al. [29] further confirmed the effectiveness of *R. communis* extracts against MRSA and *Escherichia coli* isolated from human surgical wound infections, supporting its clinical relevance. In addition, Hajrah et al. [30] used transmission electron microscopy to show that *R. communis* extracts disrupted the cell membranes of *Escherichia coli* and *Klebsiella oxytoca*, suggesting cell wall damage as a likely antibacterial mechanism. Complementing these findings, Kebede and Shibeshi [12] reported notable antibacterial and antifungal activities against *Escherichia coli* and *Candida albicans*, organisms frequently involved in polymicrobial SSI infections. Together, these studies reinforce the broad-spectrum antibacterial efficacy of *R. communis* and its potential as a natural adjunct or alternative therapy for SSI management.

Nanoparticle-facilitated studies have expanded the pharmacological applications of *R. communis*. Silver nanoparticles prepared using leaf extracts exhibited high enzyme inhibition, cytotoxicity, and enhanced antimicrobial activity [6]. Similarly, gold nanoparticles synthesized from *R. communis* extracts induced caspase-3-mediated apoptosis in human colon cancer cell lines, demonstrating the plant's multiplicity beyond its role in infection prevention [7]. Multiple independent studies confirm *R. communis*’s efficacy against resistant strains. Methanolic leaf extracts showed inhibition zones of 25 mm and 11 mm against MRSA and *Pseudomonas aeruginosa*, respectively [30]. In earlier studies, electron microscopy showed that bacteria treated with the extract had structural damage [34]. These outcomes are consistent with prior research showing that chemicals derived from Ricinus leaves inhibit MRSA [19].

*R. communis* has shown pharmacological action in wound healing, dental conditions, and skin infections. In arthritic models, it exhibits anti-inflammatory properties in addition to antiviral activities, primarily against SARS-CoV-2 [1,2]. Additionally, the plant is effective against *Gluconobacter oxydans*, a common cause of oral infections, suggesting that it could be used in oral formulations [35]. Furthermore, combining *R. communis* with other botanical extracts or nanomaterials has been shown to yield synergistic effects. It has been reported that combination therapy, which includes *R. communis* and two other plant extracts, is more successful than monotherapy [19]. These interactions may help delay the onset of resistance and lower the necessary dosages.

Recent investigations further support the antibacterial relevance of *R. communis* against MDR pathogens. Studies using metal nanoparticles synthesized from *R. communis* extracts have demonstrated enhanced antibacterial and antifungal efficacy, as well as improved stability and biocompatibility [6,7,16]. Updated pharmacological evaluations also highlight the plant’s broad-spectrum activity and its growing consideration in integrative antimicrobial strategies [18,22]. Moreover, research on plant-based combination therapies demonstrates that *R. communis* extracts, when used alongside other botanical agents, can produce synergistic effects, thereby reducing the emergence of resistance and improving efficacy [19]. These advancements align with the World Health Organization’s 2023-2024 surveillance data, which indicate an alarming rise in resistant SSI pathogens, including MRSA, ESBL-producing *Escherichia coli*, and *Klebsiella pneumoniae* [34]. The accumulating evidence reinforces R. communis as a promising candidate for the development of next-generation plant-derived antimicrobials [6,7,16,18,19,22].

Table 2 states that *R. communis* exhibits broad-spectrum antimicrobial activity, with methanolic extracts showing the highest efficacy against common SSI pathogens.

**Table 2:** Summary of key studies on the antimicrobial activity of Ricinus communis against SSI pathogens MRSA: methicillin-resistant *Staphylococcus aureus*,* *SSI: surgical site infection, MDR: multidrug-resistant

Study	Extract type/solvent	Tested microorganisms	Main findings
Abew et al. (2014) [3]	Methanolic leaf extract	*Escherichia coli*, MRSA	Strong inhibition zones; methanol most active
Kebede and Shibeshi (2022) [12]	Ethanolic and aqueous leaf extracts	*Escherichia coli*, *Candida albicans*	Significant antibacterial and antifungal activity
Voleti et al. (2022) [29]	Methanolic extract	*Staphylococcus aureus*, *Escherichia coli* (SSI isolates)	High activity; supports wound-healing potential
Hajrah et al. (2018) [30]	Ethanolic extract	*Escherichia coli*, *Klebsiella oxytoca*	Microscopy showed cell membrane disruption
Linima et al. (2023) [16]	Nanoparticle formulation	MDR pathogens	Enhanced antimicrobial efficacy
Donkor et al. (2023) [19]	Polyherbal combination	Mixed bacterial strains	Synergistic effect vs. single extracts

Discussion

This review highlights the growing significance of *R. communis* as a potential phytotherapeutic agent for the management of SSIs and is supported by international research. Research has demonstrated that *R. communis* leaf extracts have strong antibacterial activity against SSI infections, with methanol preparations yielding the best results. Gram-positive organisms are generally more sensitive to plant-derived phytochemicals than Gram-negative ones, according to reports of inhibitory ranges of 23 mm against *Staphylococcus aureus* and 19 mm against *Escherichia coli* [12,30].

These literature-based findings are consistent with the global research landscape. Other studies have reported comparable inhibition ranges, such as those of Voleti et al. [29], wherein strong activity of the methanolic extract was observed against *Staphylococcus aureus* and *Escherichia coli* in surgical wound isolates. Similar to the results of Lopes et al. [28], Hajrah et al. [30] have also shown impressive antimicrobial activity of *R. communis* extracts across numerous solvent compositions, which underscores its potential as a broad-spectrum agent. Recent investigations further reinforce this trend, highlighting that *R. communis*-based metal nanoparticles and polyherbal formulations exhibit enhanced efficacy against MDR bacteria, including MRSA and ESBL-producing *Escherichia coli* [6,7,16,18,19,22]. Collectively, the existing evidence demonstrates activity against bacteria frequently associated with SSIs, reinforcing the growing recognition of *R. communis* as a valuable plant-based antimicrobial source. These findings further support the previously reported efficacy of *R. communis* against MDR strains, including *Staphylococcus aureus* and *Escherichia coli*, which exhibit established resistance profiles. Recent global surveillance data from the World Health Organization (2023-2024) also indicate an alarming increase in resistance among common SSI pathogens, strengthening the rationale for investigating plant-derived antimicrobials [34].

Given the growing risk of MRSA and Enterobacteriaceae that produce ESBLs in SSIs [5,33], the demonstrated efficacy of *R. communis* extracts warrants consideration as an adjuvant or alternative antibacterial approach [35]. Furthermore, published studies have reported that the observed activity against *Pseudomonas aeruginosa* aligns with international findings on this pathogen's inherent resistance [30]. Comparative studies with other ethnomedicinal plants, such as *Azadirachta indica* (neem) and *Aloe vera*, indicate that *R. communis* exhibits similar or superior inhibitory profiles against Gram-positive bacteria, reinforcing its therapeutic potential among herbal antimicrobials [18,19].

Although these results are encouraging, limitations exist. Most existing research has focused primarily on in vitro models, and there remains a lack of in vivo verification assessments of efficacy, safety, or pharmacokinetics. Furthermore, systematic and meta-analytic reviews of herbal antimicrobials for SSI management (e.g., Cochrane-style reviews) consistently highlight methodological gaps, small sample sizes, and lack of clinical validation: issues that apply equally to current *R. communis* studies [36]. These constraints limit the direct clinical extrapolation of current findings. It is proposed that animal models, expanded microbiological examinations, and clinical trials be utilized in the future to explore the therapeutic potential of *R. communis* in greater detail. Standardized extraction protocols, comparative clinical evaluation, and toxicity profiling should also be prioritized to ensure reproducibility and safety. Based on accumulated global reports, *R. communis* exhibits a wide range of antimicrobial activity, particularly against Gram-positive SSI agents [37,38]. With appropriate standardization, formulation development, and rigorous clinical trials, this herb can be integrated into evidence-based infection management initiatives, particularly in regions disproportionately affected by AMR and SSIs.

Safety, toxicology, and pharmacokinetics

Cytotoxicity Profiles

A significant concern with herbal drugs is safety. *R. communis* demonstrates a favorable cytotoxicity profile when extracted from non-seed parts. The leaf extracts, which are typically used for antibacterial purposes, lack the highly toxic protein ricin found in seeds [10]. According to previous studies, minimal cytotoxicity in mammalian cells was reported when silver nanoparticles were synthesized from leaf extracts, suggesting a wide safety margin [6].

In vitro evaluations reported in the literature confirm that most leaf-derived formulations are safe at concentrations effective for antibacterial action. For instance, butanol fractions were shown to inhibit cancer cell proliferation while maintaining biocompatibility with normal cells [10].

However, the presence of ricin in seeds has raised concerns. Ricin acts by inhibiting ribosomal function, leading to multiorgan failure if ingested [1]. Fortunately, therapeutic applications typically avoid seed extracts for this reason. Emphasis has been placed on the importance of proper purification and selection of plant parts to ensure safe formulation [7].

Bioavailability and Delivery Challenges

Despite its bioactivity, *R. communis* suffers from typical phytochemical limitations: poor aqueous solubility, low absorption, and rapid metabolism. These challenges reduce bioavailability, making it difficult to translate the in vitro efficacy reported in earlier studies to in vivo therapeutic success [15]. Crude extracts may require large doses for systemic effects, which is impractical and potentially unsafe.

Nanoformulation has emerged as a promising solution. Previous research indicates that phytochemicals encapsulated into liposomes, polymeric nanoparticles, or conjugated to metallic surfaces demonstrate improved absorption, stability, and targeted delivery [6,7]. Ricinus-based nanomaterials have achieved higher bioactivity at lower concentrations, supporting nanoformulation as an effective strategy [19].

In related applications, *R. communis* has also been employed in phytoremediation studies. In these contexts, absorption challenges were addressed with biostimulants, and similar principles could inform pharmaceutical delivery systems [24].

Regulatory and Standardisation Concerns

A significant barrier to the clinical adoption of *R. communis*-based therapeutics is the lack of standardized extraction protocols. Extraction yields and phytochemical content vary significantly depending on the plant source, geographical origin, and solvent system used [28]. This variability complicates universal dosage recommendations and consistent toxicity assessment.

There is an urgent need for regulatory oversight to define Good Manufacturing Practice (GMP) protocols for botanical drugs. International organizations must collaborate to develop reference standards for key bioactives such as ricinine and gallic acid [28]. Analytical fingerprinting methods like high-performance liquid chromatography (HPLC), Fourier transform infrared spectroscopy (FTIR), or nuclear magnetic resonance (NMR) spectroscopy have been recommended to ensure quality control of herbal formulations [13].

Additionally, safety regulations must address the presence of allergenic or toxic components, such as ricin, in seed-derived extracts. Bioassay-guided fractionation and targeted extraction can help mitigate this risk. The adoption of ISO and WHO-GMP guidelines for herbal formulations will enhance the credibility of *R. communis*-based pharmaceuticals and support their integration into evidence-based medicine [10]. Comparable findings have been reported in regional studies, as summarized in Table 3.

**Table 3:** Preclinical evidence on antibacterial and cytotoxic effects of Ricinus communis MRSA: methicillin-resistant *Staphylococcus aureus, *AgNPs: silver nanoparticles, ESBL: extended-spectrum β-lactamase, HPLC: high-performance liquid chromatography, NMR: nuclear magnetic resonance, MIC: minimum inhibitory concentration, MBC: minimum bactericidal concentration

Study region	Application/model	Key outcome	Reference
Ethiopia	Antibacterial (in vitro, leaf extract)	Inhibition of *Escherichia coli*, *Candida albicans*	[12]
India	MIC/MBC against resistant strains	MIC: 1.5–4 mg/mL; ESBL inhibited	[30]
Nigeria	Cardioprotection and antibacterial synergy	Dual benefit of leaf extract	[8]
Saudi Arabia	Electron microscopy of MRSA	Bacterial cell lysis observed	[31]
South America	Gold nanoparticle-assisted anticancer study	Induced apoptosis in HT29 and SW480	[7]
India	Synergistic effects with herbal combinations	Enhanced inhibition with 3-extract mix	[19]
Pakistan	Low cytotoxicity of AgNPs from leaf extract	Biocompatible and effective antimicrobial agent	[6]
Global	Phytochemical standardization recommendations	Call for HPLC/NMR-based fingerprinting	[13]

Limitations

Although this review compiles recent evidence on the antimicrobial efficacy of *R. communis*, most available studies are limited to in vitro and animal models. Clinical data confirming its safety, pharmacokinetics, and therapeutic applicability remain scarce. Additionally, variability in extraction methods and phytochemical composition across studies may influence reproducibility. The narrative design of this review may also carry inherent selection bias toward positive findings.

Future directions and research gaps

Despite the growing body of in vitro and preclinical evidence supporting the antimicrobial and therapeutic potential of *R. communis*, several critical research gaps remain. To realize its full pharmacological value and therapeutic utility, comprehensive translational efforts are essential, encompassing clinical validation, advanced formulation development, and expansion of antimicrobial testing across broader biological targets.

Clinical trials needed

The most significant limitations of current *R. communis* research are the predominance of in vitro studies. Although disc diffusion assays and MIC evaluations have provided important preliminary data, the pharmacokinetics, biodistribution, metabolism, and systemic toxicity of *R. communis* extracts remain largely unexplored in vivo [6,31,36]. Well-designed animal models are essential for evaluating not only antibacterial efficacy in complex biological systems but also immunomodulatory, hepatotoxic, and nephrotoxic risks at therapeutic doses.

Clinical trials are another critical milestone. To date, no randomized controlled trials have assessed the efficacy of *R. communis* extracts or derivatives in human subjects for the treatment of infections or other conditions. Future clinical studies should focus on patient populations with antibiotic-resistant infections, such as MRSA or ESBL-positive SSIs, for which phytotherapeutics could offer adjunct or alternative treatments [22]. Informed by pharmacological evaluations in animal studies, early-phase trials can help define safe dosage ranges, treatment windows, and potential contraindications.

Formulation development

Another essential research direction involves developing pharmaceutical formulations using *R. communis* bioactives. Most studies use crude extracts, which are impractical for clinical use due to batch variability, instability, and inconsistent bioavailability. There is a need for standardized formulations, such as creams, gels, sprays, or injectable solutions, that deliver defined concentrations of active constituents, including ricinine, gallic acid, and kaempferol [28].

These formulations should also be tested for their compatibility with commonly used antibiotics. Synergistic formulations may help overcome microbial resistance and reduce the effective doses of synthetic drugs, thereby minimizing side effects. Previous studies have shown promise in combining herbal extracts with antibiotics, and *R. communis* could be a strong candidate for such combinatorial therapies [37].

Broadening microbial spectrum

Current investigations primarily focus on aerobic bacterial pathogens. However, SSIs and other healthcare-associated infections often involve polymicrobial communities, including anaerobic bacteria and fungi. Thus, expanding the antimicrobial spectrum of *R. communis* through testing against fungal species (e.g., *Candida albicans*, *Aspergillus* spp.) and anaerobes (e.g., *Bacteroides fragilis*) is essential [3,36].

Also, multi-pathogen models that replicate real-life infection dynamics, such as biofilm formation, immune evasion in hosts, and co-infection, are essential for determining the true therapeutic potential of *R. communis*. In vitro systems such as organoids or ex vivo tissue cultures and in vivo infection models should be implemented to gain a more comprehensive view of the pharmacodynamics of its active compounds.

## Conclusions

The collective global evidence demonstrates that *R. communis* possesses significant antibacterial properties relevant to the management of SSIs. Published studies indicate that methanolic leaf extracts exhibit strong inhibitory activity against major pathogens, including *Staphylococcus aureus*, *Escherichia coli*, and *Klebsiella pneumoniae*, highlighting their potential to combat AMR. Gram-positive organisms, particularly *Staphylococcus aureus*, are reported to be more susceptible, suggesting their value against MDR strains like MRSA. The plant’s moderate activity against Gram-negative and ESBL-producing organisms further supports its role as a complementary agent where antibiotics fail. This review uniquely integrates ethnobotanical, phytochemical, and microbiological evidence, emphasizing the translational potential of *R. communis* in SSI management. By consolidating findings across diverse regions and experimental approaches, it provides a coherent framework linking traditional use to modern antimicrobial strategies. However, literature underscores the need for standardized extraction protocols, pharmacokinetic studies, and clinical validation. *R. communis* thus remains a promising, safe, and cost-effective phytotherapeutic candidate for modern infection control and evidence-based medicine.
